# Built environment interventions and physical activity levels: A systematic review

**DOI:** 10.7705/biomedica.6113

**Published:** 2022-05-01

**Authors:** Susana Barradas, Diego Lucumí, Deivis Nicolás Guzmán-Tordecilla, Jeremy Young, Diana Pinzón

**Affiliations:** 1 Escuela de Gobierno Alberto Lleras Camargo, Universidad de los Andes, Bogotá, D.C., Colombia Universidad de los Andes Universidad de los Andes Bogotá, D.C. Colombia; 2 Facultad de Ciencias Sociales y Humanas, Universidad Externado de Colombia, Bogotá, D.C., Colombia Universidad Externado de Colombia Universidad Externado de Colombia Bogotá, D.C. Colombia; 3 Departamento de Administración, Pontificia Universidad Javeriana, Bogotá, D.C., Colombia Pontificia Universidad Javeriana Pontificia Universidad Javeriana Bogotá, D.C. Colombia; 4 Grupo de Salud Ambiental y Laboral, Instituto Nacional de Salud, Bogotá, D.C., Colombia Instituto Nacional de Salud Bogotá, D.C. Colombia

**Keywords:** built environment, physical activity, health promotion., entorno construido, actividad física, promoción de la salud.

## Abstract

**Introduction:**

Non-communicable diseases are the leading cause of death worldwide and physical activity is a key preventive strategy to reduce them. There is a relationship between the built environment and the practice of physical activity, but little evidence as to whether those built environment interventions not initially designed for promoting physical activity actually have an impact on promoting the behavior.

**Objective:**

To identify whether such built environment interventions were able to change physical activity in adults.

**Materials and methods:**

We conducted a systematic review of interventions targeting modifications to the built environment changes in urban areas.

**Results:**

Out of 5,605 articles reviewed, only seven met our inclusion criteria. The seven studies found higher levels of physical activity after the interventions.

**Conclusions:**

We recommend greater specificity regarding the study design, the timeline of interventions implementation and post-intervention measurements, as well as the use of more objective measures. Finally, we point out the need to make more explicit the mechanisms of change related to the interventions assessed.

## Resumen

**Introducción.:**

Las enfermedades no transmisibles son la principal causa de muerte en todo el mundo y la actividad física es una estrategia preventiva clave para reducirlas. Hay una relación entre el entorno construido y la práctica de actividad física, pero poca evidencia de si las intervenciones no diseñadas en principio para promoverla, realmente tienen un impacto en ese sentido.

**Objetivo.:**

Determinar si tales intervenciones en el entorno urbano pudieron cambiar la práctica de actividad física en adultos.

**Materiales y métodos.:**

Se hizo una revisión sistemática de las intervenciones que apuntaban a modificar el entorno construido en zonas urbanas.

**Resultados.:**

De 5.605 artículos considerados, solo siete cumplieron con nuestros criterios de inclusión y en todos ellos aumentó la actividad física después de la intervención.

**Conclusiones.:**

Se recomienda que el diseño del estudio, el cronograma de implementación de las intervenciones y las mediciones posteriores sean más específicas, de manera que las medidas obtenidas sean más objetivas. Asimismo, se argumenta la necesidad de hacer más explícitos los mecanismos de cambio relacionados con las intervenciones evaluadas.

Currently, non-communicable diseases (NCD) are the leading cause of death in the world, responsible for 38 million of the 56 million deaths recorded in 2012, i.e., 68% of the total deaths worldwide ^1^. The majority of deaths reported as a cause of NCD occurred in the working-age segment of the population and in low and middle-income countries ^1^^,^^2^. Specifically, the main four groups of diseases responsible for 80% of all NCD-related deaths are cardiovascular diseases, cancers, chronic respiratory diseases, and diabetes ^3^. For the period 2011 to 2025, it has been estimated that low- and-middle-income countries would have an economic loss of US $ 7 billion derived from NCD, far exceeding the annual cost of interventions aimed at reducing the prevalence of NCD (US $ 11.200 million) ^1^.

The promotion of physical activity (PA) is among the multiple interventions proposed for the reduction of morbidity and mortality of NCD (between 20 and 30%) ^1^^,^^3^^-^^5^. According to the World Health Organization ^1^^,^^2^, for countries to increase PA levels in their population and thus have a positive effect on the reduction of morbidity and mortality related to NCD, an intersector collaboration strategy is needed including transport, urban planning, recreation, sports, and education sectors.

There is evidence that suggests a relationship between the built environment and health behaviors such as PA ^6^^,^^7^. Specifically, the characteristics of the built environment can promote or inhibit PA ^6^^,^^7^. For example, the aesthetic and security infrastructure of neighborhoods is related to the frequency of physical activities such as walking and cycling ^8^. Neighborhoods with adequate pedestrian infrastructure, illumination, green spaces, and walls free of graffiti have had positive effects on the practice of PA ^9^^,^^10^. Similarly, it has been shown that recreational and non-recreational facilities (cafes, grocery stores, food stores, schools, and other services) are positively associated with active transportation ^9^^,^^10^. This suggests that even those spaces that have not been specifically built to promote PA have a relationship with its practice.

Although various studies and systematic reviews have demonstrated a strong relationship between built environments and PA ^9^^-^^11^, they do not clearly show whether environments not initially designed for promoting PA actually have an impact on such promotion. The sustainable objective goals adopted by the United Nations in 2016 have also pointed out the importance of sustainable cities and communities. Since the world is increasingly urbanized (today, 3.5 million people live in cities) and it is expected that by 2030 60% of the population will live in urban areas ^12^, it is essential to understand their role in health. Therefore, clarifying how those environments influence the practice of PA would help to encourage an inter-sector approach to promoting it.

Besides, synthesizing information on whether urban planning interventions whose main goal was not PA promotion, regardless of later measurements of PA variables or proxies, can contribute to consolidating the evidence on built environments and PA. This will also contribute to the body of knowledge related to evidence-based urban development, which can directly or indirectly have an impact on the prevalence of NCD. We checked the systematic review of the Guide to Community Preventive Services ^13^ and found that it applies only to the EUA. In this context, our goal was to determine whether built environment interventions not initially designed to promote PA were able to change PA or PA proxies in urban areas.

## Materials and methods

### Search strategy

This systematic review was conducted according to the PRISMA criteria ^14^. The search for articles took place in December 2016 in five databases: MEDLINE, Web of Science, EMBASE, ProQuest, and LILACS selected for a greater understanding of the sample and to cover as many articles as possible. Search terms used were: “intervention” OR “natural experiment” AND “physical activity OR exercise OR walking OR cycling” OR “commute mode walking OR cycling” “active commute” OR “mode of travel” OR “proportion of trips” OR “active travel” OR “travel behavior” OR “active transport” OR “connectivity” AND “built environment” OR “built environment interventions” OR “infrastructure” OR “urban planning” OR “urban interventions” OR “transportation intervention” OR “public transportation” OR “transport infrastructure” OR “urban regeneration” OR “urban revitalization” OR “housing projects” OR “green space” OR “land use” OR “lighting” OR “traffic lights” OR “roads’’

### Exclusion and inclusion criteria

The search included only papers on adult populations in English, Spanish, or Portuguese. To track the most recent trends studies, the search was limited to papers published between 2000 and 2016. We chose this period because, after the year 2000, there was increased recognition of the relation between built environment interventions and health behaviors including PA ^15^. Also, at the beginning of the new century, ecological models shed light on the role of the environment in PA promotion ^15^^,^^16^.

We only included those papers describing interventions not primarily designed to promote PA but measuring at least one outcome related to this behavior before or after the intervention. In this sense, we included those studies related to transportation or built environments designed for and available to the general population. For the purposes of this study, physical activity was defined as any muscular movement requiring energy expenditure of moderate or vigorous intensity including sports, active recreation, play, wheeling, walking, or cycling ^17^. We included articles with PA self-reported measures (including walking) and we also accepted PA proxies such as commute mode walking, active commute, mode of travel, the proportion of trips, active travel, and travel behavior. Likewise, a built environment was defined as any physical environmental characteristics in a community that could make physical activity easier or more accessible ^18^.

### Selection of studies

**Figure 1:**
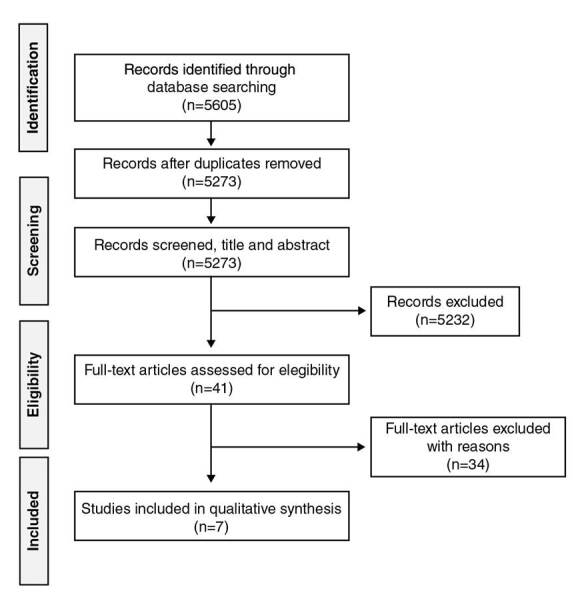
PRISMA flow diagram

We used three different filters in the selection of studies: the exclusion of duplicated articles; the review of articles and abstracts by four trained researchers with English and Spanish reading skills, one of whom also had Portuguese reading skills, to evaluate the inclusion and exclusion criteria, and the review by pairs of researchers of those articles whose inclusion was previously agreed. Possible discrepancies on whether to include a paper or not were solved by a third reviewer of the team based on the inclusion and exclusion criteria previously established. Figure 1 shows the flow diagram of the search performed in databases.

### Data extraction

Every paper included was then reviewed and categorized following these characteristics: year, language, country/region, type of study, area of knowledge, type of intervention, type of population and participants’ age, physical activity and other outcomes, effect estimate of PA, and main results (figure 1).

## Results

The results were organized as follows: First, we present the number of initial records identified in the databases and the final number of articles included in the sample. Second, we identify and describe the study design of the papers reviewed. Third, we describe the type of population or participants in the studies reviewed and the type of PA measures used in each paper, and, finally, the mechanisms underlying the interventions when this information was available.

## Sampling

A total of 5,605 records were identified through the initial database search. After excluding duplicates, 5,273 records were screened by title and abstract and 5,232 were excluded because they did not meet the inclusion criteria; the remaining 41 articles were assessed for eligibility, and seven were included in the final sample (figure 1).

### Studies design

All the studies identified were on interventions not primarily designed to promote PA as defined in the inclusion criteria. Three of them were presented as natural experiments ^19^^-^^21^, one as a prospective cohort study ^22^, one as a two-wave study ^23^, and another one as a quasi-experimental study inside a cohort study ^24^. One of the studies did not specify the research design used ^25^ but based on the information provided in the methods section, we assumed that it corresponded to a longitudinal design with pre- and post-intervention assessments (table 1).

**Table 1:** Summary of studies assessing urban interventions’ effect on unplanned physical activity outcomes

Citation	Data collected	Study design	Recruitment strategy	Demographics	Intervention	Physical activity outcome
Prins, *et al.*, 2016	Cambridge, (UK). Data collected between 2009 and 2013	Quasi-experimental (natural experiment)	Recruitment at workplace using a combination of strategies: emails, leaflets, recruitment stands	N = 469 M age = 43.9 (SD=10.8) Female = 66.4% Urbanicity: urban = 65.9%; rural = 34.1%. Education: lower than degree level = 25.4% degree level = 74.6%	Construction of new transport infrastructure that connects towns and villages in the northwest of Cambridge with the Cambridge Science Park, the city center and the Cambridge Biomedical Campus	The new routes increased the use of cycling. Among all the measurements, the increase was of 1.42, 95% CI = 0.86, 1.97).
Morrison, *et al*., 2004	Glasgow, (Scotland) Data collected between 2009 and 2013	Quasi-experimental (prospective cohort study)	Postal questionnaire surveys using household addresses from a commercial data company	N = 750 (15 years and older)	The intervention consisted in a traffic calming scheme in the main road of an urban housing estate in Glasgow.	Subjective measure of physical activity showed higher levels in walking and cycling behaviors after intervention; 20% of the participants reported to walk more in the area after the intervention. Also, 3.8% reported to cycle more in the area, 12.5% allow children to walk more, 11.6% allow children to cycle more, and 11.8% of the participants allow children to play out more in the area.
Sun, *et al*., 2014	Hong Kong, (China). Data collected between 2012 and 2013	Quasi-experimental (natural experiment)	Email recruitment of students from the university campus	N = 169 M age =18.7 (SD = 1.2) Female = 55%	A restructuring process was carried out within the campus of the Chinese University of Hong Kong: Changes in land use, pedestrian network, population density, and campus bus services schedules.	Results showed that changes in the built environment led to higher levels of walking physical activity. Changes in exposure to pedestrian network increased walking (β = 0.895; p < 0.001). Changes in the use of recreational buildings (located further away) and exposure to increased population density were related to an increase of walking distance (β = 0.187; p < 0.001).
Wells, *et al*., 2008	Georgia, Alabama, and Florida (US). Data collected between 2003 and 2006	Quasi-experimental (natural experiment)	Women in the beneficiaries’ neighborhoods of the Habitat for Humanity program. List of names provided by the local program organization	N = 32 M age = 38 Annual income = 16.425.75 USD Average body mass index = 32.09 kg/m2 Overweight or obese = 82% Education: High school graduate = 81%	Two neighborhoods were relocated to neotraditional communities and conventional suburban neighborhoods.	After the relocation, the number of steps in neotraditional neighborhoods were higher (62.207 steps/week) when comparing with suburban neighborhoods (58.617 steps/ week), but not statistically significant (p = 0.6). However, when looking for race differences, African-American women walked less (50,320 steps/week) in comparison with non-African Americans (70.504 steps/week) and this difference was significant (p = 0.013). Also, household size predicted higher number of steps per week (5,600 more steps; p = 0.008).
Hong, *et al.,* 2016	California (US). Data collected between 2012 and 2013	Quasi-experimental (two-wave study)	Addresses in the area of the study were purchased from a commercial database Invitation letters were sent to households in the area	N = 73 M age = 38 Male = 39% Race (white: 26%; black: 56%) Education: some years of college = 29%, bachelor = 34% Employment status: not employed = 44%	Construction of a new light rail line	In the second statistical model using total walk trip counts and the interaction term between treatment and baseline MVPA, being in the treatment group was associated with higher levels of moderate to vigorous PA (MVPA) at follow-up (β = 9.29; p = 0.06). However, when looking for the intersection between treatment and baseline MVPA, those effects were attenuated (β = −0.34; p = 0.06).
Panter, *et al*., 2016	Cambridge (UK). Data collected between 2009 and 2012	Quasi-experimental	Recruitment at workplace using a combination of strategies: Newspaper advertisements, posters, flyers by means of corporate email, staff newsletters, recruitment stands	N = 469 M age = 44 (SD = 11.1) Women = 66.5% Education: degree level education: 74.8%; less than degreelevel education: 25.2%. Urban-rural status: urban 67.3%; town 17.1%; village 15.6% Weight status: overweight or obese: 33.9%	Opening of a new transport infrastructure “Cambridgeshire Guided Busway”	Positive effect of the exposure to the busway. Greater amount of weekly cycling (relative risk ratio = 1.34; 95% CI = 1.03, 1.76). Also, more time spent in active commuting (relative risk ratio = 1.76; 95% CI = 1.16, 2.67) only for those participants with less active commuting at baseline. Participants living closer to busway showed more cycling and less walking, and as the distance from the busway increased, this relationship was reversed.
Brown, *et al.*, 2015	Salt Lake, Utah (US) Data collected between 2012 and 2013 (one week before and after the intervention)	Quasi-experimental	Participants were recruited door to door	N = 537 M age = 41.1 (SD = 0.74) Female = 51% Hispanic = 25% College graduates = 37% Married = 46%	Street intervention to extend a light-rail line	Intervention was associated with PA levels assessed with accelerometers. Former riders showed a decrease in PA levels in comparison with never-riders (t = −3.30; p = 0.001). New users of the light rail line performed more PA in comparison with never-riders (t = 2.72; p = 0.007).

### Study population characteristics

The studies included a total of 1,947 participants in the different interventions ranging between 70 and 537 while one of them gathered information from 750 households ^22^. Regarding population characteristics, two studies recruited employees of a certain area of interest ^21^^,^^24^, three recruited people from a local community ^22^^,^^23^^,^^25^, one recruited university students ^20^, and another one, low-income African American women ^19^.

### Type and focus of interventions

All the interventions evaluated PA levels associated with changes in the built environment such as transport infrastructure, traffic calming schemes, and street interventions, among others (table 1). Four studies evaluated specifically a transport infrastructure Intervention ^21^^,^^23^^-^^25^, another one, a traffic-calming scheme ^22^, and two other studies evaluated built environment characteristics such as land use, pedestrian networks, and street network patterns ^19^^,^^20^.

### PA and PA proxies measures

The papers reviewed used different approaches to measure PA (table 1): walking minutes or distance ^19^^,^^20^^,^^22^^-^^24^, cycling minutes or distance ^21^^,^^22^^,^^24^, total minutes per week of total moderate to vigorous PA (hereafter MVPA) ^23^^,^^25^, and total minutes per week of moderate to vigorous recreational PA ^24^. These measures were obtained either objectively or through self-report. Objective measures of walking were collected through accelerometry ^23^^,^^25^ or pedometer ^19^. Subjective measures were collected using validated scales ^24^ in a survey with questions regarding the weekly time dedicated to PA ^21^^,^^23^ or by asking directly for perceived differences in PA levels as a result of the intervention ^20^^,^^22^.

All the measures in the studies reviewed were taken before and after the interventions, though in one of them, the measure was taken right after the intervention. In the other studies, the follow-up measurements were made at different times after the interventions had become effective: three months later ^20^, five to seven months later ^23^, and one year later ^21^. The other studies did not give exact information in this regard ^19^^,^^21^^,^^22^^,^^24^ and, although they mentioned the range of time over which the data were collected, it was not clear how much time elapsed between the implementation of the intervention and the follow-up.

### Mechanisms underlying the interventions

Regarding the underlying mechanism of interventions, we used the Behavioral Change Theory (BCT) as the theoretical, methodological, and analytical foundation to explain changes in PA behavior and proxies. Two studies have made explicit reference to the theories of change, specifically the Theory of Planned Behavior (TPB) ^21^^,^^23^, the Health Belief Model, and the Ecological Model ^23^. On the other hand, one of the studies measured variables that have been integrated into behavior theories and models (e.g., perceptions and attitudes) still without making any explicit reference to theory ^22^. The study by Prins, *et al*. ^21^, applied theory frameworks ^26^ including an open reference to TPB whose variables were measured and analyzed in conjunction with the results for physical activity and changes in the built environment. In the case of Hong, *et al*. ^23^, it seems the study was more informed by TBP but they did not apply it sufficiently during the study ^26^. Regarding the study by Morrison, *et al*. ^22^, although there's some level of theory application, it failed to account for a specific theory in its theoretical framework.

## Discussion

To our knowledge, this is the first systematic review analyzing whether built environment interventions not initially designed to change PA could actually achieve it in urban areas. As systematic reviews in this field are usually interested in interventions designed to impact PA levels, our focus on interventions not designed to change PA is, we believe, a unique contribution to the literature. A better understanding of how built environments can influence the practice of PA should lead to the use of an inter-sector approach in public health, particularly in the efforts to promote PA in urban areas. As noted above, our synthesis of evidence on these urban planning interventions as affecting PA can contribute to consolidating our knowledge regarding built environments and PA performance in urban areas.

All the studies reviewed found higher levels of PA and PA proxies after the interventions. These results appear to be consistent with other authors’ findings regarding the influence of built environments on health behaviors, particularly in PA ^6^^,^^7^^,^^27^^,^^28^. The results indicate that the kind of interventions conducted in our sample can be particularly useful in increasing walking, cycling, total MVPA, and recreational MVPA in communities.

Three methodological aspects are relevant in our evaluation of the selected studies. First, although the studies reviewed used a quasi-experimental design, not all of them described the study design exhaustively and one failed in mentioning it at all ^25^. We recommend that future studies should be more specific about the research design and how it is carried out. Second, these studies are not specific enough regarding the timeline of interventions’ implementation and when post-intervention measures were obtained. In this sense, it is very important to report the time-lapse of follow-up since the findings of a study can refer to the period of observation or to the moment of the event, which, in this case, was the intervention ^29^. In this sense, we suggest longer follow-up times, like those in the articles we reviewed where the evaluation was carried out immediately after the implementation of the interventions. Finally, more than half of the studies reviewed used only self-reported measures, which are not as reliable as objective measures and present a high risk of bias ^30^. We suggest the use of more objective measures, for example, the use of accelerometry or pedometers could be used along with subjective measures. Another potentially valuable possibility is the use of Ecological Momentary Assessment (EMA), which allows for obtaining information on PA behavior and its correlates in real-time ^31^.

Another important observation was that the studies did not discuss enough how these interventions impact underserved communities and disadvantaged groups. In our sample, only one study discussed this issue with reference to low-income African American women ^19^. Besides, we did not find any studies in low- or middle-income countries that fit our inclusion criteria of pre- and post-intervention measures on the effect of urban interventions not originally designed to promote PA that could have had a potential effect on this behavior (e.g., BRT, cables, electric stairs, etc.). This evidence is especially useful in economically disadvantaged countries since they tend to experience a disproportionate burden of NCD explained in part by PA low levels.

Another important issue was the authors’ understanding of the mechanisms of change related to the interventions assessed along with the theoretical decisions guiding measure selection. An adequate understanding of the mechanism of action behind the interventions would allow explaining behavior changes related to specific interventions ^32^. Most of the studies, however, did not make an explicit reference to a theory of change and among those that did, only one tested the theory using variables proposed in the TPB in their pre- and post-intervention measurements ^19^. In this sense, we consider that authors’ conceptual frameworks of intervention mechanisms should be made more explicit. Also, the theoretical decisions guiding the selection of measures should be stated more clearly and in greater detail.

According to the World Health Organization ^33^, it is essential to adopt a “Health in All Policies” approach to help improve populations’ health and health equity. Accordingly, we recommend that interventions in the built environment take into account aspects to benefit and promote the practice of physical activity, especially in low- and middle-income countries.

We did not find any study in languages other than English and none were carried out in Latin America and the Caribbean region, which we believe is a limitation of the study. Although we wanted to focus on interventions that were not initially designed to change PA, we only used health sciences databases because we were analyzing the PA outcome measure. Our expectation was to include studies in low-income and minority communities, but only one of them explicitly referred to the inclusions of participants in this category and, therefore, it was not possible to draw generalizations for such populations.

As for the strengths of the study, it is worth mentioning that we included interventions from fields other than health whose purpose was not the promotion of healthy behaviors such as PA but can nonetheless be effective in promoting them. Additionally, our interdisciplinary and multilingual team broadened the review scope with abstracts in English, Spanish, and Portuguese. Our findings showed that built environment interventions not designed to promote PA are potentially effective in encouraging this behavior.
